# Assessment of sites of marrow and extramedullary hematopoiesis by hybrid imaging in primary myelofibrosis patients

**DOI:** 10.1002/cam4.835

**Published:** 2016-08-12

**Authors:** Mario Ojeda‐Uribe, Olivier Morel, Constantin Ungureanu, Christophe Desterke, Marie‐Caroline Le Bousse‐Kerdilès, Hatem Boulahdour

**Affiliations:** ^1^ Service d'Hématologie Clinique et Unité de Thérapie CellulaireHôpital E MullerGHR Mulhouse Sud‐AlsaceMulhouseFrance; ^2^ Pôle d'ImagerieCHU BesançonFrance; ^3^ INSERM UMS33Hôpital Paul‐BrousseVillejuifFrance; ^4^ Université Paris Sud 11VillejuifFrance; ^5^ INSERM U1197Hôpital Paul‐BrousseVillejuifFrance; ^6^ EA 4662‐Nanomedicine Lab, Imagery and TherapeuticsHôpital J MinjozCHU BesançonFrance

**Keywords:** Extramedullary hematopoiesis, hybrid imaging, primary myelofibrosis, radionuclides, SPECT, spleen

## Abstract

We investigated noninvasive procedures by hybrid imaging to assess the sites of active or inactive hematopoiesis in patients with primary myelofibrosis (PMF). To this end, we used two radionuclides, technetium 99m (^99m^Tc) and indium 111‐chloride (^111^In‐Cl_3_), coupled with single‐photon emission tomography/computed tomography (SPECT/CT). We studied five patients with PMF and one with secondary myelofibrosis (MF). The classical pattern of lower fixation of both tracers at the axial skeleton where the myelofibrotic process occurs and the reactivation of sites of active hematopoiesis at the distal skeleton were confirmed. Coupling both radionuclides to SPECT/CT imaging allowed for more precise visualization of the sites of extramedullary hematopoiesis as those observed in the spleen and liver. Splenic high uptake of ^111^In‐Cl_3_ coupled with SPECT/CT represents a pathognomonic feature of PMF. We conclude that, the hybrid imaging procedures that we studied might constitute an alternative noninvasive method for the screening of the whole‐body marrow and, by this way, to assess the impact of targeted therapies in PMF patients in whom it is well known that the distribution of the hematopoietic active areas is disturbed. Hybrid imaging could also be useful for diagnostic purposes in cases of early PMF or in suspected cases of myelofibrosis secondary to polycythemia vera or essential thrombocythemia.

## Introduction

The dynamic relationship between the skeleton and bone marrow (BM) hematopoietic niches throughout life implies that clinical entities may arise from hematopoietic stem cells (HSCs) and/or BM microenvironment abnormalities 1, 2. Two HSC niches are described: the endosteal/osteoblastic niche, where HSCs interact with osteoblasts, and the perivascular niches, where HSCs are close to endothelial cells, mesenchymal stromal cells, and pericytes surrounding the BM vascular structures 1, 2. The relationship between HSCs, their niches, and the trabecular bone is disrupted in myeloproliferative neoplasms, such as primary myelofibrosis (PMF) or secondary MF (sMF) 3, 4. Signal transduction abnormalities in PMF‐HSCs can be driven by somatic mutations, such as those involving the genes *JAK2*,* CALR*, or *MPL* 5.

Primary myelofibrosis is associated with extramedullary hematopoiesis (EMH), usually with splenomegaly and occasionally, hepatomegaly. Several tracers are used in nuclear medicine to assess EMH and BM hematopoietic activity. Colloids labeled with ^99m^Tc show the reticuloendothelial system 4. After intravenous (IV) injection, ^99m^Tc nanocolloids are rapidly cleared from the plasma. The physiological uptake in normal adult humans is as follows: liver, 70%; spleen, 10%; and BM, 15–20%. ^111^In‐Cl_3_‐transferrin scintigraphy reflects the erythropoietic activity of BM and other sites of active hematopoiesis. Following IV injection, it is rapidly coupled with transferrin and cleared from the plasma. The physiological uptake is as follows: liver, 20%; spleen, 1%; BM, 30%; and kidney, 7%. Both radiopharmaceuticals show a physiological uptake in the axial skeleton, but not in the distal skeleton [6].

Cell imaging using single‐photon emission tomography (SPECT) utilizes gamma‐emitting radiopharmaceuticals to allow two‐dimensional (2D) imaging acquisition. After reconstruction, visualization of three‐dimensional (3D) images in multiple planes is possible. It is often coupled to computed tomography (CT) (SPECT/CT) [6].

Few data exist regarding hematopoiesis assessment in PMF using hybrid imaging. We have previously shown its applicability in the EMH diagnosis 7 . In PMF, we have also used this technique for many years and because of recent developments in its molecular aspects, we consider this noninvasive procedure might allow us to assess the impact of novel targeted therapies 5.

## Material and Methods

### Study design

We retrospectively studied hybrid imaging procedures we had performed in five untreated PMF patients and in one patient with essential thrombocythemia (ET) who was developing secondary MF (Table 1).

**Table 1:** Baseline clinical and biological characteristics of the patients studied and summary of the hybrid imaging patterns observed with the two radiopharmaceuticals used

	Axial skeleton		Spleen		Liver		Distal skeleton		EMH SPECT/CT		Disease status and molecular profile
	^99m^Tc	^111^In	^99m^Tc	^111^In	^99m^Tc	^111^In	^99m^Tc	^111^In	^99m^Tc	^111^In	
P1	Fixation	Lower fixation	Hyperfixation	Hyperfixation	Hyperfixation	Fixation	Fixation	Higher fixation	Neg	Neg	PMF‐F V617F‐JAK2^pos^
P2	Fixation	Fixation	Fixation	Hyperfixation	Fixation	Fixation	Fixation	Higher fixation	Neg	Neg	PMF‐F JAK2^pos^ (exon12) CALR^neg^ MPL^neg^
P3	Lower fixation	Lower fixation	Fixation	Hyperfixation	Fixation	Fixation	Fixation	Higher fixation	Neg	Neg	PMF‐F JAK2^pos^ (exon12) CALR^neg^ MPL^neg^
P4	Lower fixation	Fixation	Fixation	Hyperfixation	Fixation	Fixation	Fixation	Higher fixation	Neg	Neg	PMF‐F V671F‐ JAK2^pos^
P5	Lower fixation	Fixation	Fixation	Fixation	Fixation	Fixation	No fixation	No fixation	Neg	Neg	Post‐TE‐MF V617F‐JAK2^pos^
P6	Very low fixation	Fixation	Fixation	Hyperfixation	Fixation	Fixation	Low fixation	Higher fixation	Neg	Neg	PMF‐F V617F‐JAK2^pos^

SPECT/CT, single‐photon emission tomography/computed tomography; EMH, extramedullary hematopoiesis; PMF, primary myelofibrosis.

These diagnostic procedures were performed according to the Helsinki declaration and approved by the local pluridisciplinary Oncology‐Hematology Committee. All patients gave their informed consent, after receiving detailed information of the benefits and risks of the procedure.

A CT was added to the routine radionuclide procedures for PMF, which is already widely used in nuclear medicine. Here, it was performed at low dose using the X‐ray tube of our hybrid device that was also employed for attenuation correction (AC). This correction helped us in the uptake assessment of deep soft structures, such as the spleen and liver.

Diagnosis of PMF and ET was made according to WHO 2008 recommendations 8. Myelofibrosis severity was based on BM biopsies assessed according to international recommendations. Interestingly, with respect to the molecular features of the PMF patients studied, two of them presented mutations in the exon 12 of *JAK2* without a previous clinical or biological history of polycythemia vera and this for at least 10 years before the diagnosis of PMF (Table 1).

Hybrid imaging procedures: two tracers were employed: (1) ^99m^Tc‐marked nanocolloid (Nanocis, IBA, Gif‐sur‐Yvette, France) scintigraphy. Whole‐body planar and focused SPECT acquisitions were performed using a dual head gamma camera (Hawkeye, GE Healthcare, Fairfield, CT, USA). The whole‐body acquisitions were made 1 and 6 h after the IV administration of ^99m^Tc nanocolloids using the following acquisition parameters: a low energy collimator, a sweep speed of 12 cm/min for the whole body, and 300 sec for static images. SPECT/CT (SPECT: 60 projections, OSEM: 2 iterations, 10 subsets, Butterworth: 0.48/10, 128 × 128 matrix; CT: slice thickness: 10 mm, 140 KV, 40 mA, 256 × 256 matrix) was also performed, and multiplanar reconstructions were made. (2) ^111^In‐Cl_3_‐transferrin scintigraphy (Covidien, Mallinckrodt Medical, Petten, The Netherlands) with SPECT/CT (60 projections; OSEM: 2 iterations, 10 subsets; Butterworth: 0.48/10; 128 × 128 matrix; CT: slices thickness: 10 mm, 140 KV, 40 mA, 256 × 256 matrix). Scintigraphy image acquisitions were made 48 and 72 h after IV administration of ^111^In‐ Cl_3_, with the following acquisition parameters: low energy collimator, a sweep speed of 10 cm/min for the whole body, and 600 sec for static images.

## Results

### Axial, distal skeleton, and splenic uptake profiles

**Figure 1:**
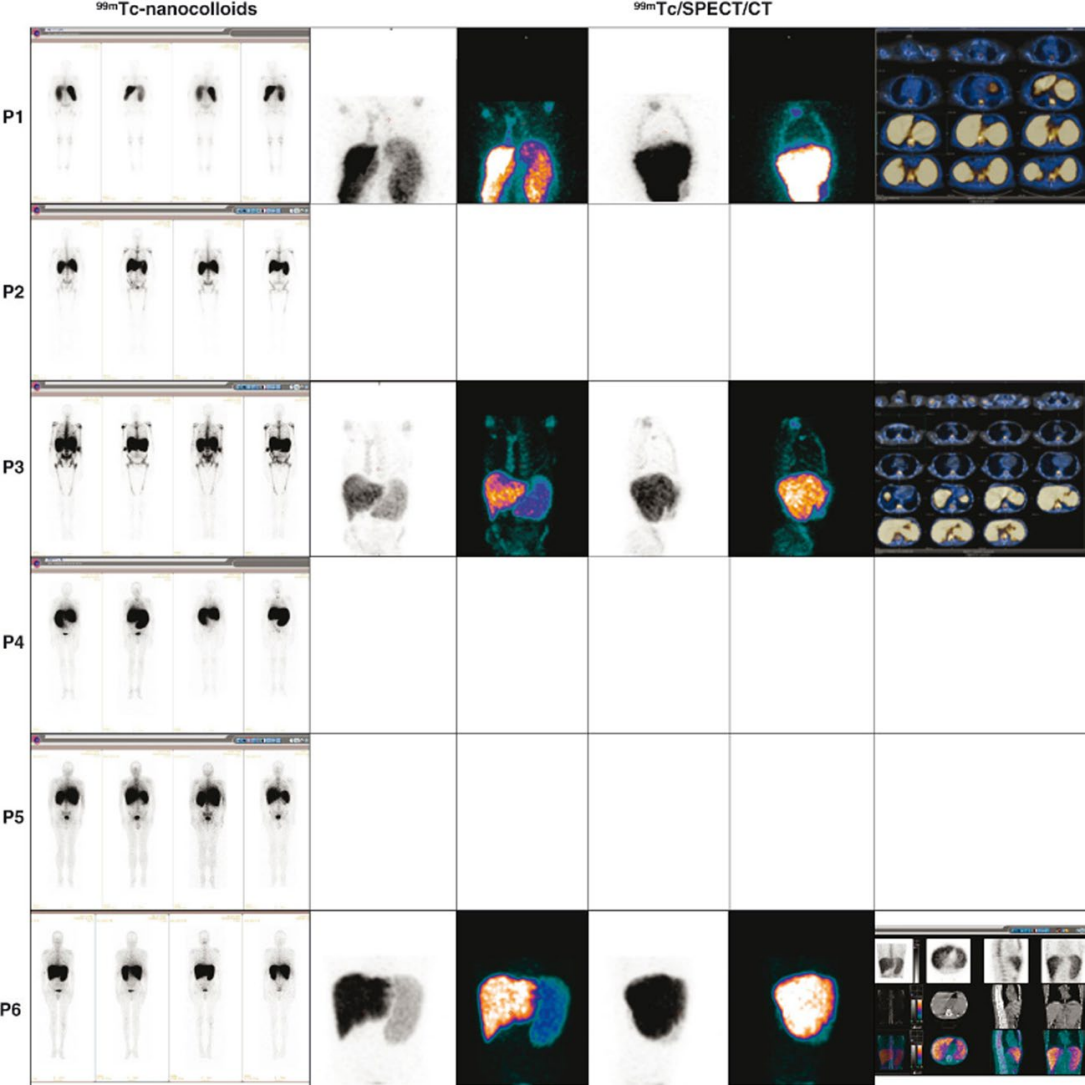
^99m^Tc‐nanocolloid (Nanocis, Iba) scintigraphy coupled to single‐photon emission tomography/computed tomography acquisitions. Representative imaging of the results is shown here: UPN1 to UPN4 and UPN6 were primary myelofibrosis patients; UPN5 was a secondary (post‐ET) MF patient.

**Figure 2:**
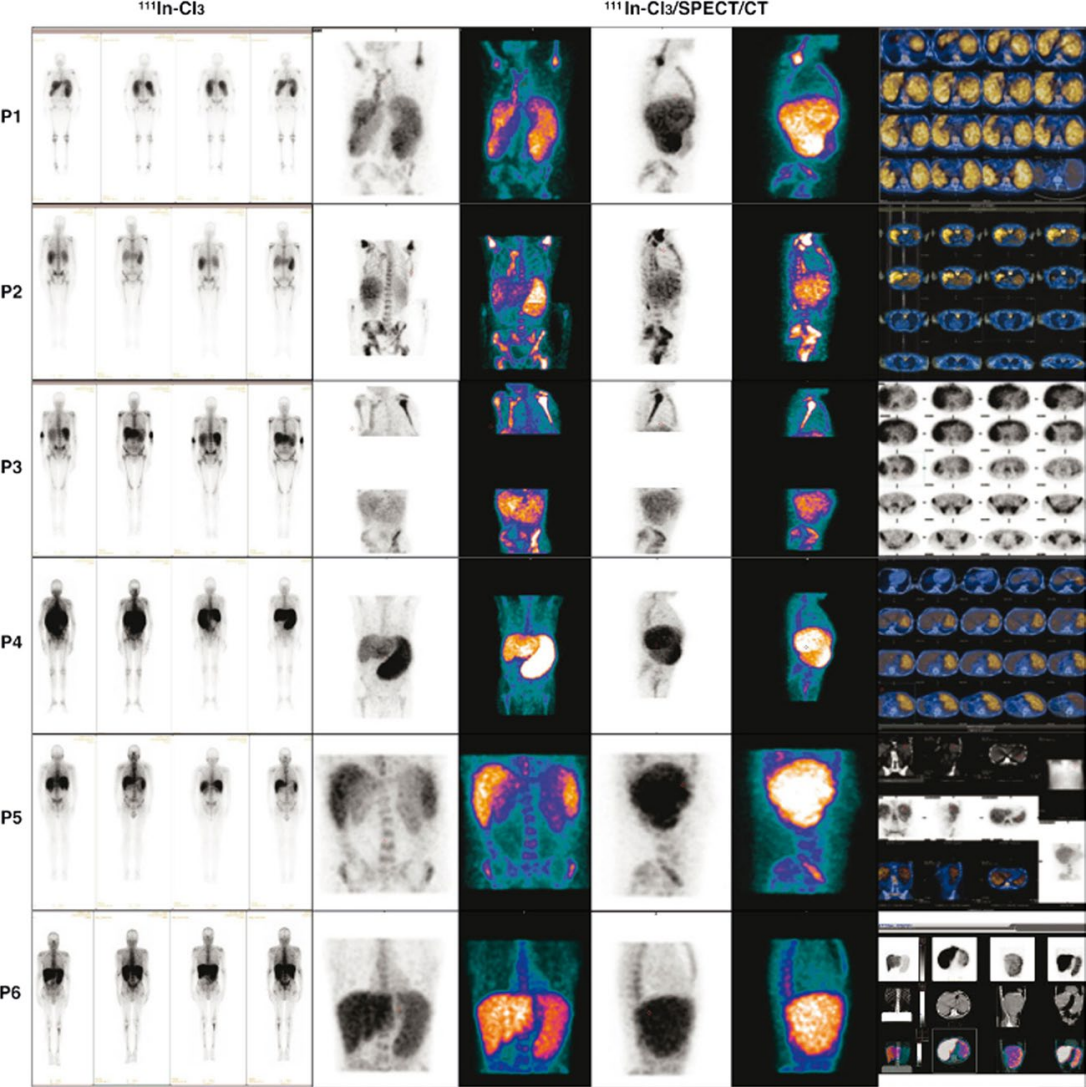
^111^In‐Cl_3_‐scintigraphy (Covidien) with single‐photon emission tomography/computed tomography acquisitions. Representative imaging of the results is shown here. UPN1 to UPN4 and UPN6 were primary myelofibrosis patients; UPN5 was a secondary (post‐ET) MF patient.

**Figure 3:**
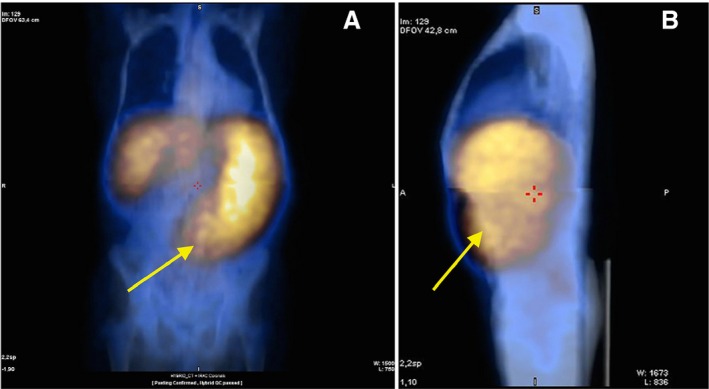
^111^In‐Cl_3_ single‐photon emission tomography/computed tomography imaging in one patient with very advanced primary myelofibrosis: (A) front view and (B) side view showing very intense uptake of the radiotracer in the spleen (yellow arrow) and almost no fixation in the backbone and other structures of the axial skeleton.

In PMF patients, we observed a lower fixation of both tracers ^99m^Tc and ^111^In‐Cl_3_ in the axial skeleton and, conversely, a higher fixation in the distal skeleton (Figs. 1 and 2; Table 1). Moreover, using hybrid imaging of ^111^In‐ Cl_3_ coupled to SPECT/CT allowed for a more detailed visualization of the extent and intensity of the EMH detected in the spleen of the PMF patients we studied (Fig. 3). In patients with advanced fibrotic process (Fig. 2, patients 1 to 4 and 6), this high splenic uptake was associated to a similar pattern of high ^111^In‐Cl_3_ fixation in the distal skeleton.

### Hepatic uptake profile

No other clear EMH localization besides the spleen was observed, except at the hepatic level. The interpretation of liver fixation was uneasy because both radiopharmaceuticals ^99m^Tc nanocolloids and ^111^In‐Cl_3_ show significant physiological uptake although more importantly in the case of ^99m^Tc nanocolloids. Regarding this last radionuclide, we observed higher hepatic fixation with ^99m^Tc‐nanocolloids in only one patient (P1) (Fig. 1). When we looked at ^111^In‐Cl_3_ distribution and intensity patterns, we observed a high liver uptake in most patients and this was concomitant to the high uptake of this radionuclide in the spleen, although some patients (e.g., P4) showed lower liver uptake intensity (Fig. 2).

### Secondary early myelofibrosis uptake profile

Interestingly, in the sMF (post‐ET) patient (P5), there was no ^111^In‐Cl_3_ hyperfixation at the distal skeleton. Conversely, we observed a significant splenic fixation with both radiopharmaceuticals and a low fixation with ^99m^Tc‐nanocolloids in the axial skeleton (Figs. 1 and 2).

## Discussion

### Axial, distal skeleton, and splenic uptake profiles in patients with advanced myelofibrosis

Regarding the splenic high uptake of ^111^In‐Cl_3_, it can be considered to be pathognomonic of PMF or sMF, because it reflects the EMH developed in this setting. In patients with advanced fibrotic process, this high splenic uptake was associated to a similar pattern of high ^111^In‐Cl_3_ fixation in the distal skeleton that also reflects the development of active hematopoiesis or at least a significant erythroblastic activity in these areas.

We consider, the more advanced the process of marrow fibrosis and HSC externalization is, the stronger is the ^111^In‐Cl_3_ splenic uptake. In conclusion, when associated with a lower fixation of this same radionuclide in the axial skeleton, the combined pattern of strong ^111^In‐Cl_3_ splenic and distal skeletal uptake was highly suggestive and pathognomonic of PMF (Figs. 2 and 3).

Some questions remain unsolved regarding the mechanisms involved in the distal skeleton recruitment or EMH reactivation in circumstances where these hematopoietic areas were progressively inactive since birth and replaced with adipose tissue 9. In adults over 25 years old, almost all the active BM (red marrow) is located in the axial skeleton and the nonactive BM (yellow marrow) is mostly located on the distal skeleton 10, 11. Yellow marrow is composed approximately of 95% fat cells whereas red marrow composition is 40% fat cells and 60% hematopoietic cells 11. Is the distal skeleton reactivation observed in PMF a consequence of HSCs that cannot seed in the physiological homing hematopoietic areas, which participate essentially in the axial skeleton in adults? Or is the fruit of HSC reactivation (and their microenvironment) already present in the distal skeleton but simply dormant? In this latter case, we do not know whether HSCs carry the same molecular abnormalities than those of the malignant PMF clone.

### Secondary early myelofibrosis uptake profile

The hybrid imaging pattern observed here evocates a shift of the active physiologic hematopoietic sites to other nonphysiologic sites following a sequence of hematopoietic reactivation starting in the spleen, followed by distal skeleton recruitment and associated with progressive hematopoietic regression in the axial skeleton (Figs. 1 and 2).

### Interest in hybrid imaging

The radiopharmaceutical patterns we observed in PMF have already been described, although without SPECT/CT 12. Hybrid imaging using these two radionuclides and SPECT/CT is a noninvasive procedure, allowing a larger and more precise visualization of the active/inactive hematopoietic areas in PMF. Moreover, regarding deep regions or overweight patients, AC can show more uptake focus, potentially hidden because of soft tissue interposition between the emitting source and detector. This can represent an alternative to BM biopsies that are limited in their investigation to the iliac crest and do not allow a whole‐body marrow screening and are also limited because of the possibility of sampling error. BM biopsies may well reflect the axial skeleton and the significant hematopoietic activity that occurs in this area in adults but they do not provide any information on distal skeleton or the liver and spleen hematopoiesis.

### Inflammatory versus myelofibrotic component of PMF

Recent studies have highlighted the interest in other noninvasive procedures, such as ^18^F‐fluorodeoxyglucose (^18^F‐FDG) positron emission tomography/computed tomography (PET/CT), to assess the inflammatory component of PMF, which is a critical player in the PMF pathophysiology 13, 14. Actually, ^18^F‐FDG PET/CT in this setting might be considered a necessary complement of the hybrid imaging techniques we studied. However, the imaging patterns in PMF of the respective radiopharmaceutical distribution are not similar. In a recent study, ^18^F‐FDG PET/CT uptake was increased in the axial and distal skeleton when the degree of marrow fibrosis was low (grade I) and was rather mild when the degree of marrow fibrosis was high (grade III) 13. The authors concluded a significant inverse correlation between the extent of the metabolically active disease (axial skeleton and distal skeleton plus spleen) and time since the diagnosis of myelofibrosis 13. This reported inverse correlation pattern with ^18^F‐FDG PET/CT assessment contrasts with the ^111^In‐Cl_3_/SPECT/CT imaging we observed in the sMF patient (P5) of our series who was at an early phase of the disease and showing a lower fixation to ^99m^Tc nanocolloids and ^111^In‐Cl_3_ at the distal skeleton. These data in early phases of myelofibrosis, can reflect a high inflammatory activity without achieving a reconversion of inactive into active BM at the distal skeleton.

On the contrary, in patients with more advanced PMF (P1 to P4 and P6, Figs. 1 and 2) high fixation patterns of the distal skeleton were observed when both techniques were compared, implying that hematopoiesis reactivation at this level is also associated with an inflammatory process 13. Moreover, age‐related changes probably need to be considered if we want to assess the importance of ^18^F‐FDG PET/CT uptake values in patients with PMF 9.

In our series, most of the patients presented significant marrow fibrosis (grade III) and, as we already mentioned, these patients with advanced disease had ^111^In‐Cl_3_ imaging patterns in favor of hematopoietic reactivation of the distal skeleton. Based on our observations, the splenic EMH that was well detected by ^111^In‐Cl_3_/SPECT/CT appears to be associated with a high inflammatory process (Fig. 3). We can obtain some evidence of this inflammatory component of PMF from ^99m^Tc nanocolloids/SPECT/CT imaging, which reflects, in part, the activity of several cell types involved in inflammation. In conclusion, on the basis of the data from our study, we observed a correlated time‐dependent pattern of the shift of active sites of hematopoiesis detected by ^111^In‐Cl_3_/SPECT/CT.

Interestingly, the imaging patterns we observed with ^111^In‐Cl_3_/SPECT/CT are quite similar to those observed with the use of another radiotracer, 3′‐18Fluoro‐3′‐deoxy‐L‐thymidine (18F‐FLT) 15. Moreover, in a few cases, hybrid imaging has been reported to be useful to detect some cases of EMH 7, 16.

In summary, and based on our preliminary results, we believe the use of hybrid imaging allowed (1) good evaluation of the extent and intensity of the sites of active/inactive hematopoiesis in PMF patients; (2) this noninvasive procedure can also be useful in challenging differential diagnosis cases of NPMs or (3) when a myelofibrotic transformation of ET or polycythemia vera is suspected. However, the interest of this technique to assess the impact of targeted therapies needs further evaluation.

## Conflicts of Interest

The authors have no conflicts of interest to disclose.
